# Clinical comparison of five anti-chlamydial antibiotics in koalas (*Phascolarctos cinereus*)

**DOI:** 10.1371/journal.pone.0236758

**Published:** 2020-07-30

**Authors:** Rosemary Booth, Sharon Nyari

**Affiliations:** 1 Australia Zoo Wildlife Hospital, Beerwah, Queensland, Australia; 2 Genecology Research Centre, University of the Sunshine Coast, Sippy Downs, Queensland, Australia; University of the Pacific, UNITED STATES

## Abstract

Chlamydiosis is the most significant infectious disease of koalas (*Phascolarctos cinereus*). It is primarily a systemic sexually transmitted disease caused by *Chlamydia pecorum* and was responsible for 46% of the 2348 koala admissions to Australia Zoo Wildlife Hospital between 2013 and 2017. Treatment of chlamydiosis in koalas is complicated by three major factors. Firstly, koalas rely on bacterial fermentation of their high fibre diet making antibiotic therapy a risk. Secondly, they possess efficient metabolic pathways for the excretion of plant toxins and potentially of therapeutic agents. Thirdly, wild koalas, often present to rehabilitation facilities with chronic and severe disease. Traditional anti-chlamydial antibiotics used in other species may cause fatal dysbiosis in koalas or be excreted before they can be effective. We compared five anti-chlamydial antibiotics, azithromycin, chloramphenicol, doxycycline, enrofloxacin and florfenicol, which were used to treat 86 wild koalas with chlamydiosis presented to Australia Zoo Wildlife Hospital under consistent conditions of nutrition, housing, husbandry and climate. Response to treatment was assessed by recovery from clinical signs, and clearance of detectable *Chlamydia* via quantitative PCR. Doxycycline was the most effective anti-chlamydial antibiotic of the five, producing a 97% cure rate, followed by chloramphenicol (81%), enrofloxacin (75%), florfenicol (66%) and azithromycin (25%). The long-acting injectable preparation of doxycycline was well tolerated by koalas when administered via the subcutaneous route, and the weekly dosing requirement is a major advantage when treating wild animals. These findings indicate that doxycycline is the current drug of choice for the treatment of chlamydiosis in koalas, with chloramphenicol being the best alternative.

## Introduction

**Fig 1:**
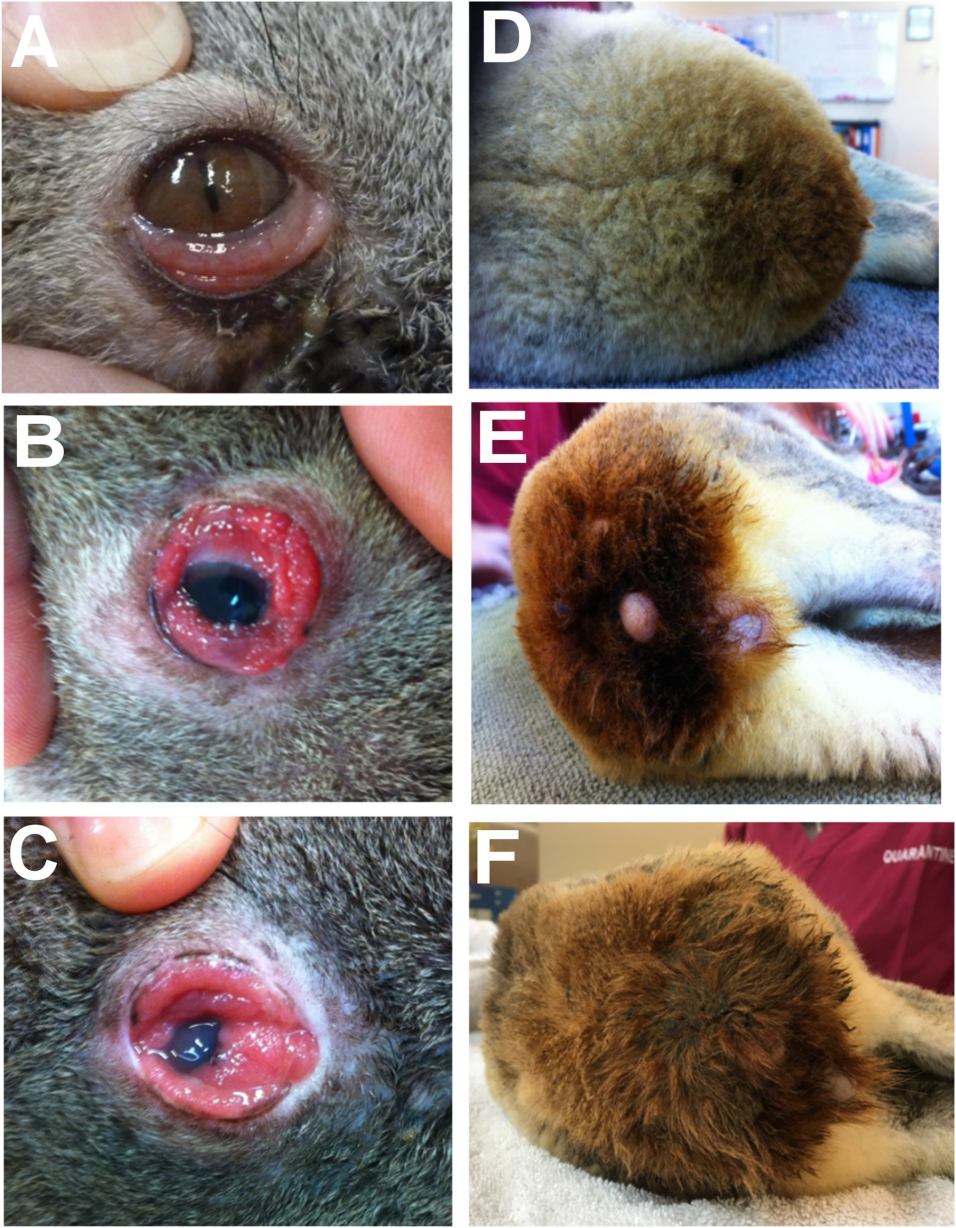
Primary clinical signs of chlamydiosis in koalas. (A-C) Mild, moderate and severe chlamydial conjunctivitis and (D-F) mild, moderate and severe urine staining of the rump fur caused by chronic dribbling of urine due to chlamydial cystitis. Equivalent to Grades 1–3.

Chlamydiosis is the most significant infectious disease of koalas (*Phascolarctos cinereus*), with a prevalence of up to 100% in some wild populations [1–3]. The major clinical signs are conjunctivitis and a brown-stained wet rump caused by chronic cystitis (Fig 1). Epidemics of ocular disease in koalas have been reported since the early 1900s [4]. In 1974 *Chlamydia psittaci* was first linked to koala keratoconjunctivitis [5] and soon after as the cause of urogenital disease in koalas [6]. In 1995 the causative organism of chlamydiosis in koalas was reclassified as two species: *Chlamydia pecorum* and *Chlamydia pneumoniae* [7].

Since the aetiology was confirmed, thousands of koalas have been treated for chlamydiosis with a range of antibiotics. As in other species, antibiotic choice is an evidence-based decision based on knowledge of pathogen sensitivity, generic and species-specific adverse effects, metabolism and excretion pathways, and the required route of administration, frequency and duration of therapy. In addition, knowledge of the pharmacokinetics of various antibiotics in koalas provides information on drug metabolism that supports decisions on dose, route, and frequency of administration. Species-specific metabolic pathways in wildlife can alter treatment regimens from those developed for humans or domestic species. Response to treatment is assessed by improvement in clinical signs, and a lack of detectable infectious organism post-treatment.

Although described as easy to treat in humans [8], treatment of chlamydiosis in wild koalas is complicated by the chronic nature of their disease at presentation and by their hindgut fermentative digestion. Some antibiotics such as oxytetracycline, which are effective against chlamydia in other species, have caused fatal dysbiosis (alteration in the caecal microbiome) due to their effect on vital tannin-protein complex-degrading enterobacteria (T-PCDE) [9]. Since the late 1980s, antibiotics such as enrofloxacin and chloramphenicol have been used with clinical success to treat chlamydiosis in koalas, while avoiding fatal impacts on gut flora. Since 2010, pharmacokinetic studies in koalas of some commonly used anti-chlamydial antibiotics have been undertaken [10–12], resulting in widespread use of chloramphenicol to treat chlamydiosis in koalas, however this drug is no longer commercially available. The reduced availability of chloramphenicol, and the evidence of occasional bone marrow hypoplasia in koalas treated with prolonged courses of this antibiotic led to this evaluation of alternative anti-chlamydial antibiotics in koalas.

The aim of this study was to compare the efficacy of five anti-chlamydial antibiotics to find the ideal therapeutic agent for wild koalas with the intention to: 1) produce a high microbial cure rate (˃90%); 2) be readily available and inexpensive; 3) cause minimal adverse effects; 4) not cause pain on injection; 5) be available in concentrations that allow small injection volumes; 6) be effective following subcutaneous or intramuscular injection; and 7) require infrequent dosing.

## Materials and methods

### Study location

The study was conducted at Australia Zoo Wildlife Hospital (AZWH), Beerwah, Queensland. The purpose of the hospital is to treat sick, injured and orphaned wildlife with the goal of returning animals to the wild fit, healthy and capable of reproducing. The study was undertaken in a clinical setting where animal welfare takes precedence over other considerations.

### Animals and allocation to treatment

All koalas presented to AZWH with ocular or urogenital signs of chlamydiosis from June 2016 to June 2017 were assessed for inclusion in this study. A total of 86 koalas, 48 males and 38 females, were suitable for inclusion based on high probability of recovery. Koalas being treated ranged in age between 1 and 10 years, as determined by tooth wear class [13]. Eligible animals were assigned to a treatment group on the basis of temperament, disease severity, body condition and history of previous antibiotic treatment. Of the 86 koalas treated, 6 required re-treatment, bringing the total number of overall antibiotic treatments to 92 (Table 1). Anxious or aggressive koalas were treated with antibiotics requiring less frequent administration. Koalas which had been treated and released previously and returned with clinical signs of chlamydiosis were not treated again with chloramphenicol. All koalas were treated and assessed at AZWH under consistent conditions of husbandry and nutrition. Koala which recovered were released within their prescribed habitat (within 5 km of point of rescue) (Table 1).

**Table 1:** Antibiotic, sex, age, date of admission and outcome of treated koalas.

Antibiotic	Koala Accession #	Male/Female	Koala Age in years	Admission Date	Outcome
**Azithromycin**	65306	Male	6	4/18/2016	Released
**Azithromycin**	65647	Female	6	5/13/2016	Deceased
**Azithromycin**	65707	Male	10	6/25/2016	Deceased
**Azithromycin**	66116	Male	6	6/18/2016	Deceased
**Chloramphenicol**	69720	Male	4	1/2/2017	Released
**Chloramphenicol**	67376	Female	3	9/23/2016	Released
**Chloramphenicol**	63513	Female	1	1/17/2016	Deceased
**Chloramphenicol**	65391	Female	5	4/26/2016	Re-treated
**Chloramphenicol**	65704	Female	10	5/20/2016	Deceased
**Chloramphenicol**	66239	Female	5	7/1/2016	Released
**Chloramphenicol**	67538	Male	4	9/30/2016	Released
**Chloramphenicol**	68349	Male	10	11/10/2016	Released
**Chloramphenicol**	68350	Female	7	11/10/2016	Deceased
**Chloramphenicol**	68352	Male	3	11/10/2016	Released
**Chloramphenicol**	68381	Male	5	11/11/2016	Released
**Chloramphenicol**	68856	Female	4	11/24/2016	Released
**Chloramphenicol**	68857	Male	7	11/24/2016	Released
**Chloramphenicol**	68858	Male	3	11/24/2016	Released
**Chloramphenicol**	68865	Female	3	11/24/2016	Released
**Chloramphenicol**	69013	Female	1	12/1/2016	Released
**Chloramphenicol**	69030	Female	7	12/1/2016	Deceased
**Chloramphenicol**	69480	Male	3	12/20/2016	Released
**Chloramphenicol**	69505	Female	10	12/22/2016	Released
**Chloramphenicol**	69716	Female	12	1/2/2017	Released
**Chloramphenicol**	69718	Female	5	1/2/2017	Released
**Chloramphenicol**	69719	Female	4	1/2/2017	Released
**Chloramphenicol**	69722	Female	4	1/2/2017	Released
**Chloramphenicol**	69798	Male	8	1/6/2017	Released
**Chloramphenicol**	69958	Female	7	1/9/2017	Released
**Chloramphenicol**	70139	Male	10	1/17/2017	Deceased
**Chloramphenicol**	70468	Female	5	2/3/2017	Released
**Chloramphenicol**	70678	Male	3	2/17/2017	Deceased
**Chloramphenicol**	71766	Male	3	4/23/2017	Deceased
**Chloramphenicol**	70908	Female	1	3/2/2017	Released
**Chloramphenicol**	71632	Male	4	4/21/2017	Released
**Doxycycline**	65391	Female	5	4/26/2016	Recovered
**Doxycycline**	65741	Female	7	5/24/2016	Released
**Doxycycline**	66415	Male	6	7/15/2016	Deceased
**Doxycycline**	66517	Male	3	7/27/2016	Released
**Doxycycline**	66991	Female	2	8/14/2016	Released
**Doxycycline**	67684	Male	7	10/6/2016	Deceased
**Doxycycline**	67696	Male	5	10/6/2016	Released
**Doxycycline**	67784	Male	4	10/13/2016	Released
**Doxycycline**	67967	Female	3	10/22/2016	Deceased
**Doxycycline**	68006	Male	7	10/24/2016	Released
**Doxycycline**	68234	Female	3	11/4/2016	Released
**Doxycycline**	68250	Male	7	11/5/2016	Recovered
**Doxycycline**	68299	Male	5	11/7/2016	Deceased
**Doxycycline**	68333	Male	4	11/9/2016	Released
**Doxycycline**	68334	Female	3	11/9/2016	Released
**Doxycycline**	68335	Male	3	11/9/2016	Released
**Doxycycline**	68757	Male	4	11/21/2016	Recovered
**Doxycycline**	68788	Male	5	11/22/2016	Recovered
**Doxycycline**	69293	Male	12	12/12/2016	Recovered
**Doxycycline**	69505	Female	10	12/22/2016	Released
**Doxycycline**	69908	Female	4	1/6/2017	Released
**Doxycycline**	70024	Female	9	1/13/2017	Released
**Doxycycline**	70195	Female	3	1/20/2017	Released
**Doxycycline**	70718	Female	4	2/21/2017	Recovered
**Doxycycline**	71123	Male	5	3/14/2017	Released
**Doxycycline**	71126	Male	7	3/14/2017	Released
**Doxycycline**	71232	Male	1.5	3/18/2017	Released
**Doxycycline**	71988	Female	3	5/7/2017	Deceased
**Doxycycline**	72021	Male	5	5/9/2017	Released
**Doxycycline**	72246	Male	3	5/27/2017	Released
**Doxycycline**	72296	Female	3	5/31/2017	Deceased
**Doxycycline**	69720	Male	4	1/2/2017	Released
**Enrofloxacin**	63513	Female	1	1/17/2016	Re-treated
**Enrofloxacin**	64241	Male	4	2/7/2016	Released
**Enrofloxacin**	64653	Female	2	2/29/2016	Released
**Enrofloxacin**	65147	Male	8	4/7/2016	Released
**Enrofloxacin**	65306	Male	6	4/18/2016	Re-treated
**Enrofloxacin**	65321	Male	7	4/19/2016	Released
**Enrofloxacin**	65375	Male	3	4/24/2016	Released
**Enrofloxacin**	65491	Male	9	4/30/2016	Released
**Enrofloxacin**	65651	Male	3	5/13/2016	Released
**Enrofloxacin**	65704	Female	10	5/20/2016	Re-treated
**Enrofloxacin**	66008	Male	12	4/20/2016	Released
**Enrofloxacin**	70048	Male	5	1/14/2017	Euthanised
**Enrofloxacin**	70195	Female	3	1/20/2017	Released
**Enrofloxacin**	70508	Female	8	2/6/2017	Released
**Enrofloxacin**	70961	Female	4	3/5/2017	Recovered
**Enrofloxacin**	71123	Male	5	3/14/2017	Re-treated
**Florfenicol**	66801	Female	5	8/20/2016	Deceased
**Florfenicol**	67156	Male	5	9/11/2016	Deceased
**Florfenicol**	67227	Male	3	9/12/2016	Deceased
**Florfenicol**	67285	Female	4	9/17/2016	Deceased
**Florfenicol**	67384	Male	6	9/24/2016	Deceased
**Florfenicol**	67572	Male	6	10/2/2016	Released
**Florfenicol**	67703	Female	4	10/7/2016	Deceased
**Florfenicol**	67784	Male	4	10/13/2016	Released
**Florfenicol**	67376	Female	3	9/23/2016	Re-treated

Re-treated animals had a positive PCR at the end of treatment with antibiotic one and required treatment with a second antibiotic to clear their chlamydial infections.

Recovered animals that were not released had insufficient vision at the end of treatment due to chronic corneal scarring caused by the Chlamydial infection.

Deceased animals were not necessarily deceased due to antibiotic side effects, as chronic Chlamydial disease is extremely debilitating.

Animals which exhibited pain on urination during the treatment trial were additionally treated with analgesics and/or anti-inflammatory drugs as indicated. This included Temgesic® (Indivior: buprenorphine 300ug/kg @ 0.01 mg/kg I/M every 8 hours as required), Rimadyl® (Zoetis: Carprofen 50 mg/ml @ 4 mg/kg loading dose followed by 2 mg/kg S/C SID for 5–7 days), or Pentosan® (Ceva: Pentosan Polysulphate 100 mg/ml @ 3 mg/kg S/C every 7 days x 4).

Koalas which did not respond to treatment, had life threatening side effects, or developed life threatening issues unrelated to chlamydiosis treatment were euthanised by sedating with intramuscular Alfaxan® (Jurox: alfaxalone 10 mg/ml) and then euthanased with intravenous Lethabarb® (Virbac: pentobarbitone 325 mg/ml) (Table 1).

Wild animals are cared for under a Department of Environment and Heritage Protection Rehabilitation permit WIRP18601117 and Department of Agriculture and Fisheries Scientific Purposes permit SUR000234. All samples were collected during normal clinical protocols.

### Selection of antibiotics and treatment regimens

The antibiotics and treatment regimens used in this study were based on recommendations for treating chlamydiosis in koalas, or domestic species and humans, if no precedent existed (Table 2).

**Table 2:** Treatment regimens for assessed anti-chlamydial antibiotics in koalas.

Antibiotic (Manufacturer)	Concentration	Dose rate	Frequency	Route	Duration	Comments	Ref
**Azithromycin (Pfizer)**	100 mg/mL	20 mg/kg	SID	IV	3 days	Diluted to a 2 mg/mL solution in sterile 0.9% saline and infused over 30 minutes in sedated koalas. Child dose rate.	[14]
**Chloramphenicol (Ceva)**	150 mg/mL	60 mg/kg	SID	S/C	28 days	Ready to use; rotated between sites; dose rate previously used to successfully treat chlamydiosis in koalas.	[15]
**Doxycycline LA (Vetafarm)**	50 mg/mL	5 mg/kg	Every 7 days	IM or S/C	28 days	Diluted 50:50 in saline; less tissue reaction if given s/c, rotated between sites; small animal dose rates.	[16]
**Enrofloxacin (Bayer)**	50 mg/mL	10 mg/kg loading dose; then 5 mg/kg	SID	S/C	28 days	Diluted 50:50 in saline; rotated between sites; small animal dose rates.	[17]
**Florfenicol (Merck)**	300 mg/mL	20 mg/kg	Every 2 days	S/C	28 days	Ready to use, domestic animal dose rate.	[18]

LA = long-acting formulation; SID = once daily, S/C = Subcutaneous, IV = Intravenous, IM = Intramuscular.

### Topical treatment of conjunctivitis

Chlamydiosis is a systemic infection and conjunctivitis responds to systemic antibiotic treatment with or without topical treatment. Particularly during the first week of treatment, ocular therapeutic support is recommended. During this trial ocufloxacin eye drops (Ocuflox®) and chloramphenicol eye ointment were used in conjunction with systemic antibiotic therapy. Anti-inflammatory therapy with dexamethasone (Maxidex^®^) or Chloroptosone ointment (provided corneal ulceration had been excluded) or with ketorolac tromethamine (Acular^®^) is also beneficial when inflammation is severe. Subconjunctival injection with a long-acting corticosteriod (Depo-Medrol^®^) proved useful to reduce severe hyperplastic conjunctivitis in chronic cases, and judicious surgical debridement of excessive proliferative tissue at the end of systemic treatment is occasionally indicated.

### Clinical assessment and sample collection

Each koala received a full clinical examination under isoflurane anaesthesia on admission to AZWH. This examination included monitoring temperature, heart rate and respiration; measuring packed cell volume and total protein of a blood sample; ultrasound evaluation of the reproductive and urinary tracts; microscopic evaluation of an abdominal paracentesis sample; and other diagnostic tests as indicated during the examination. Clinical signs of chlamydiosis were graded as *mild*, *moderate* or *severe* on the basis of the examination and clinical progress was then evaluated daily. Copan® 160C aluminium shaft minitip dry swabs were used to collect epithelial cells from the conjunctival, urogenital tract and rectum of koalas for quantification of the chlamydial load via qPCR. Prior to June 2016, Clearview® tests were used to confirm Chlamydial infections. Swab samples were collected at time of admission (time–point 0), and at the end of treatment. Swab samples were stored frozen at -20°C until analysed. All koalas received an end of treatment visual check and a full clinical examination under anaesthesia 3 weeks after the end of treatment to collect post treatment PCR swabs and carry out pre-release procedures (tagging and microchipping). End of treatment sample collection at 3 weeks post treatment was selected as this is the optimal time in humans to avoid false positives from residual nucleic acid fragments [14]. Koalas received an additional evaluation under anaesthetic whenever there was a clinical concern during treatment.

### Bodyweight

All koalas were weighed during their initial examination and at regular intervals throughout treatment. In uncomplicated cases the frequency of weight measurements was every 7 days, or up to daily if there were clinical concerns during treatment. Body weight for each individual was charted to monitor trends during treatment.

### DNA extraction and *Chlamydia pecorum* quantification

Swab samples collected from the ocular, urogenital and rectal sites of koalas, were thawed at room temperature and added to 1.5 mL sucrose phosphate glutamate at pH 7.4 (0.2 M sucrose, 3.8 mM potassium phosphate monobasic, 8.6 mM disodium phosphate, 4.9 mM glutamic acid). Swabs were vortexed for 3 mins then 1 mL solution was removed and centrifuged at 18 000 rpm for 20 mins. The pellet was re-suspended in 50 μL TE buffer and heated at 95ºC for 10 mins. DNA extraction was then performed using QIAmp DNA mini kit (Qiagen), as per manufactures instructions, with the exception that the proteinase K digestion was incubated at 56ºC for 12 hours. Extracted DNA was then screened for the presence of *C*. *pecorum* and subsequent chlamydial load using quantitative real-time PCR (qPCR). The forward primer: 5’ AGTCGAACGGAATAATGGCT 3’, and the reverse primer: 5’ CCAACAAGCTGATATCCCAC 3’ targeted a 204 bp fragment of the *C*. *pecorum* 16S rRNA gene. Each PCR reaction contained 5 μL of DNA template added to a mastermix containing, 1x Quantitect SYBR Green (Qiagen), 0.5 μM of each forward and reverse primer and molecular grade water making a final volume of 20 μL per reaction. For PCR amplification, there was an initial denaturation at 95ºC for 15 mins, followed by 35 cycles of 94ºC for 15 secs, 57ºC for 15 secs and 72ºC for 30 secs. All reactions were performed in duplicate and samples of ˃ 100 copies/μL were considered positive. All reactions were carried out on a Rotor-Gene Q 5-plex HRM platform (Qiagen).

### Clinical pathology

For a subset of the treated koalas, a haematological screen (Procyte) and a 13-parameter biochemistry panel (Idexx) were evaluated at admission, during the treatment period if any clinical concerns were noted, and at the end of treatment.

### Effect on gut flora

The impact of the antibiotics on gut flora was evaluated via faecal smears if abnormalities in faecal pellets, body weight decreases or inappetence were observed during treatment. Koalas with faecal candida or changes in normal faecal flora were treated with additional therapeutic agents, as indicated.

### Detection of persistent infection

Successful treatment of infection was assessed via qPCR results on ocular, urogenital and rectal swabs taken at 3 weeks after treatment. Quantitative-PCRs were considered positive at a threshold of >100 copies/μl. Treatment success was defined as negative PCR (<100 copies/μl) at all three sites and recovery from clinical signs of disease. Treatment failure was defined as persistent positive PCR (>100 copies/μl) at any of the 3 sites, or persistent clinical signs of disease. Successfully treated koalas were released back into the wild. Those animals which tested positive underwent a further 28 day course of treatment with a different antibiotic (usually doxycycline as more evidence became available).

### Improvement in clinical signs

In cases of conjunctivitis, improvement was assessed by resolution of erythema, swelling, epiphora, corneal inflammation and pain (evidenced by squinting). In cases of cystitis, improvement was assessed by absence of pain on urination (evidenced by not vocalising when urinating), resolution of incontinence (dry fur around the cloaca and rump), reduction in bladder wall thickness at ultrasound and the absence of blood or excess sediment in the urine. Orange brown staining of the fur of the rump persists for many weeks after the resolution of cystitis as stained fur is gradually replaced by new white hairs.

## Results

### Clearance of infection

*C*. *pecorum* loads, greater than a million copies/μL, were detected at admission. After the initial 28 day treatment, 15 koalas continued to have a positive qPCR result from at least one of the three sample sites, requiring further treatment. Urogenital and rectal swabs were more frequently positive after treatment than were conjunctival swabs (Table 3). None of the antibiotics were 100% effective (Table 4). Mortality occurred in all treatment groups, except enrofloxacin. Mortality was not necessarily the consequence of antibiotic therapy, as chronic chlamydiosis is an extremely debilitating disease in koalas and co-infection with other pathogens can also alter the outcome.

**Table 3:** Koalas with persistent positive qPCR results after antibiotic treatment for chlamydiosis.

Antibiotic	Koala ID	Pre-Treatment			3 weeks post treatment end		
		Conjunctiva	UGS	Rectal	Conjunctiva	UGS	Rectal
**Azithromycin**	65707	Clearview +ve			0	0	20 000
**Chloramphenicol**	65391	Clearview +ve			0	2 000	4 000
**Chloramphenicol**	69505	1 325	18 300	180 000	0	0	1 503
**Chloramphenicol**	69720	3 300	0	0	0	5 780	0
**Chloramphenicol**	68433	10 000	2 926	NR	0	518	0
**Chloramphenicol**	69718	242	0	NR	300	0	0
**Chloramphenicol**	63513	Clearview +ve			0	900	900
**Doxycycline**	65391	0	2 000	4 000	0	240	0
**Enrofloxacin**	70195	0	21 000	63 000	0	44 457	20 920
**Enrofloxacin**	65683	Clearview +ve			400	2 200	1 000
**Enrofloxacin**	71123	516	0	0	585	0	0
**Enrofloxacin**	70718	0	8 677	16 487	0	190	16 620
**Florfenicol**	67376	0	350	700	0	125	120
**Florfenicol**	67227	15	5 500	2 500	0	165 000	550
**Florfenicol**	67285	Clearview +ve			100	250	200

qPCR = quantitative polymerase chain reaction; Conj = conjunctival swab; UGS = urogenital system swab; Rectal = rectal swab, NR–no result.

**Table 4:** Efficacy of antibiotic treatment in koalas assessed 3 weeks after the end of treatment.

Antibiotic	Number treated	Treatment failures (%)^a^	Treatment Success (%)^b^
**Azithromycin**	4	3 (75)	1 (25)
**Doxycycline**	32	1 (3)	31 (97)
**Chloramphenicol**	31	6 (19)	25 (81)
**Enrofloxacin**	16	4 (25)	12 (75)
**Florfenicol**	9	3 (33)	6 (66)
**Total**	92	17 (18)	75 (82)

^a^Treatment failure was defined as persistent positive conjunctival and/or urogenital and/or rectal quantitative polymerase chain reaction tests (qPCRs), and/or persistence of clinical signs

^b^treatment success was defined as negative conjunctival *and* urogenital *and* rectal qPCRs 3 weeks after treatment, and recovery from clinical signs. Negative PCR was defined as less than 100 copies/μl.

### Clinical response

The five antibiotics produced varying degrees of improvement in the clinical signs of chlamydiosis. Doxycycline was the most effective anti-chlamydial antibiotic tested, achieving both clinical recovery and infection control in 97% of cases. Side effects included those typical of broad-spectrum antibiotic therapy in koalas: occasional weight loss, depression, candidiasis, dysbiosis and typhlocolitis. In addition, marked pain on intramuscular injection was overcome by diluting the long acting preparation of doxycycline 50:50 with sterile saline prior to subcutaneous injection.

Chloramphenicol was also effective with 81% of treated individuals being qPCR negative at the end of treatment (Table 4). Side effects also included weight loss, depression, candidiasis, dysbiosis and typhlocolitis but also bone marrow hypoplasia. Florfenicol, an analogue of chloramphenicol known to cause less bone marrow suppression in domestic animals, produced poor results in koalas on the treatment regimen used in this study, being effective in 66% of cases treated (Table 4). It was also associated with pronounced weight loss, inappetence and depression. On this basis, use of this drug was discontinued after treating 8 koalas. Enrofloxacin achieved both clinical improvement and elimination of chlamydial DNA in 75% of the koalas in this treatment group (Table 4).

Azithromycin in koalas was trialled using the regime developed for children, with the advantage of a 3-day course of treatment. The azithromycin treatment group experienced the most severe gastrointestinal side effects, depression and body weight loss, and only one of 4 koalas treated survived to release. However, all koalas selected for treatment with azithromycin had very severe clinical signs of chlamydiosis and all showed a rapid and dramatic improvement in their clinical signs during the first 14 days of therapy. Another disadvantage of azithromycin is that it is painful even on intravenous injection; therefore, it was diluted and given intravenously, over 30 minutes, to sedated or anaesthetised koalas (Table 2). These factors made azithromycin the least effective (Table 4) and least convenient of the five antibiotics used. Its effectiveness as an anti-chlamydial antibiotic suggests that with advances in supportive care, in particular maintaining the caecal microbiome, there may be a role for this antibiotic in specific cases.

The efficacy of the five antibiotics were evaluated on their ability to meet seven criteria developed to represent an ideal therapeutic antibiotic (Table 5). Doxycycline performed the best meeting six of the seven criteria.

**Table 5:** Efficacy of five antibiotics against seven criteria.

	Azithromycin	Chloramphenicol	Doxycycline	Enrofloxacin	Florfenicol
**Effective >90%**	No	Almost	Yes	No	No
**Available and affordable**	Yes	No	Yes	Yes	Yes
**Minimal Side effects**	No	Yes though worse than D and E	Yes	Yes	No
**No pain on injection**	No, intravenous injection painful even when diluted and given over 30 minutes. Done under GA.	Yes	No	Yes	Yes
****			Painful if given i/m as manufactured		
			Less painful if diluted 1:1 in saline and given s/c		
**Small injection volume**	No	Yes	Yes	Yes	Yes
**Absorbed s/c or i/m**	No	Yes	Yes	Yes	Yes
**Infrequent Dosing**	Yes	No	Yes	No	No

i/m = intramuscular, s/c = subcutaneous, GA = general anaesthetic.

### Bodyweight

In the majority of cases, treatment for chlamydiosis was associated with loss of bodyweight, particularly during the first week when wild animals are adapting to temporary captivity. Many of the treated koalas experienced a less than 5% drop in bodyweight during week 1 of treatment. However, koalas treated with azithromycin lost an average of 9% body weight (range 4.4 to 12.4%) during the first week of treatment.

### Clinical pathology

In the subset of koalas in which blood was analysed, the haematological and biochemical parameters measured were within the normal range for koalas in all treatment groups. Abnormalities detected were primarily in electrolytes, PCV/TP or white cell differentials, but were not consistently associated with any treatment group.

### Effect on gut flora

During treatment with any broad-spectrum antibiotic, koalas may develop gastrointestinal pathology, primarily candidiasis, diarrhoea, caeco-colic dysbiosis (altered gut flora) or typhlocolitis (inflammation of the caecum and colon). The use of chloramphenicol was frequently associated with gastrointestinal candidiasis, usually at about 2 weeks, into the 28-day treatment. Diarrhoea is a frequent intermittent occurrence in koalas and may be associated with leaf species ingested or as a side effect of antibiotic therapy. Dysbiosis, as assessed by faecal smear, was observed in koalas in all treatment groups. Typhlocolitis can be a life-threatening condition in koalas and was detected occasionally in the koalas in this study and likely to be associated with the impact of broad spectrum antibiotics on essential microbe populations in the caecum and colon.

## Discussion

In this hospital-based study of the treatment of chlamydiosis in koalas, doxycycline and chloramphenicol were the most effective antibiotics of the five tested and produced good clinical outcomes. The microbiological cure rates for doxycycline and chloramphenicol were 97% and 81%, respectively. All antibiotics used were effective in some of the koalas treated, however none were effective in all animals.

Side effects from the use of broad-spectrum antibiotics are common in koalas, which depend on hindgut fermentative digestion of their high-fibre diet. Hence, management of dysbiosis is an important component of treatment for chlamydiosis in koalas. In this study, oral and intestinal yeast infections from about day 14 of treatment were common. Gastro-intestinal candidiasis can be managed in koalas with nystatin or amphotericin B. Caeco-colic dysbiosis can be managed with the oral administration of fresh caecal contents harvested from recently deceased koalas (stored at 4⁰C for up to 14 days). More detailed analysis of the gut associated side effects for doxycycline and chloramphenicol in koalas are in process.

Doxycycline was the only antibiotic tested that met most (6 of 7) of the criteria for an ‘ideal’ anti-chlamydial agent for koalas. The only criterion not satisfied was that of non-painful injection. Intramuscular injection of the long-acting 50 mg/ml solution is painful, which produces a vigorous patient reaction and can cause transient lameness and, occasionally, swelling. Hence, we modified the treatment regimen by diluting the oily preparation (50:50 in sterile saline) immediately before subcutaneous injection. The subcutaneous route for Doxycycline proved clinically effective in this study and pharmacokinetics for this route in this species is underway.

Chloramphenicol is a highly effective and well-tolerated broad-spectrum antibiotic, but it can cause blood dyscrasias [19] and is no longer commercially manufactured in the injectable form because of human susceptibility to this side effect. Chloramphenicol has been used to successfully treat chlamydial conjunctivitis and cystitis in koalas at the dose and regimen used in this study [20]. Pharmacokinetic studies support that the current dosage regimen is probably effective in koalas [10]. Gastro-intestinal side effects are common in koalas and can be managed as discussed above. Bone marrow hypoplasia is also a common side effect of chloramphenicol in other species [21] and has been identified in koalas at the Australia Zoo Wildlife Hospital, but could also be associated with exogenous koala retrovirus (KoRV) infection.

Compared with chloramphenicol, florfenicol, a thiamphenicol derivative, is significantly more active in vitro against many pathogenic strains of bacteria. Molecular structural differences improve efficacy, reduce toxicity and reduce bacterial resistance; *Chlamydia* species are susceptible [22]. In koalas, pharmacokinetic studies suggest that florfenicol does not persist beyond 24 hours when given by intravenous injection at 10 mg/kg and does not reach the mean inhibitory concentration (MIC) for *Chlamydia* [12]; the dose regimen used in this study was 20 mg/kg given subcutaneously every second day for 28 days based on the dose rate used in livestock. On this treatment regimen, florfenicol produced a low cure rate compared to the other antibiotics trialled, but did nevertheless eliminate detectable chlamydial DNA from 6 koalas.

When indicated by culture and sensitivity, enrofloxacin has been used in koalas to successfully treat a range of bacterial infections, including skin and pulmonary infections, and has been given by the intravenous, subcutaneous, oral and inhalation routes. However, poor oral bioavailability at dose rates up to 20 mg/kg was reported by Griffith *et al*. (2010) [10]. The authors speculated that oral enrofloxacin is unlikely to be effective against *Chlamydia* in koalas. The recommended dose rate used in this study was extrapolated from small animal dose rates: 10 mg/kg subcutaneous loading dose followed by 5 mg/kg subcutaneously daily for 28 days. Enrofloxacin was only moderately effective in this study (75% success rate) but caused no major side effects or mortality. Despite pharmacokinetic evidence, enrofloxacin has produced good clinical results in koalas with one hospital releasing more than 828 koalas over a 10 year period that responded to treatment, particularly those with conjunctivitis. This was prior to the availability of qPCR to confirm therapeutic effectiveness. Enrofloxacin has a good uptake into infected cells and this clinical improvement may be associated with a reduction of the chlamydial pathogen load, and the supportive care that the koalas receive once admitted to hospital [23]. However, the effectiveness of enrofloxacin in the treatment of *Chlamydia*-infected koalas has also been shown to fail, with koalas remaining positive after treatment of up to six-months [24].

The US Centers for Disease Control and Prevention (CDC) recommend azithromycin or doxycycline to treat human chlamydial infections; both drugs have a 95% microbial cure rate in humans. Azithromycin has been used to treat chlamydial infertility in women and conjunctivitis in babies [25–27]. Azithromycin was only used in koalas with very severe disease because there was an unpublished report of mortality in a koala treated with it. Severe chronic chlamydiosis contributed to the high mortality in this group. Daily monitoring of body weight in this group suggested the most severe gut associated side effects. Despite this, one koala which had not been cleared of his clinical chlamydiosis after a 28 day course of chloramphenicol and a 28 day course of enrofloxacin, made a full recovery and was released after treatment with 3 intravenous injections of azithromycin.

### Limitations

Successful treatment of chlamydial infection is more likely if initiated early in the disease process. Wild koalas are admitted to wildlife hospitals when they have either become so sick as to be on the ground, or when their clinical signs are easily detected while they are in a tree, and they are rescued for treatment. The duration of their infection is not known at the time of admission. There is variation in the susceptibility to the disease, with some koalas in populations with a high prevalence of chlamydiosis living and breeding without contracting the disease. Co-infection with KoRV may also modulate immune responses to chlamydial infection and contribute to the severity of the disease within individuals.

Attempts to match animals in the treatment groups in terms of age, sex and severity of disease were abandoned due to the random presentation of cases from the wild. For practical reasons, koalas were allocated into treatment groups according to temperament, severity of disease, body condition and history of previous antibiotic treatment failure. For example, koalas which were considered likely to die from their chlamydial disease without rapid intervention were treated with azithromycin. Thus, the evaluation of azithromycin was compromised by its use in koalas with a lower-than-average chance of recovery. Also, animals allocated to the doxycycline treatment group included the more aggressive or anxious koalas because this drug had the lowest dosing frequency; these animals experienced higher levels of stress from their confinement in captivity, but this drug performed the best in this trial. Further, koalas which had not responded to 28 days of therapy with either chloramphenicol (n = 6) or enrofloxacin (n = 4) were then treated with doxycycline, which may have made the residual infection easier to treat although some of the qPCRs were still very high at the commencement of doxycycline therapy.

The impact of antibiotics on gut flora could be more thoroughly assessed by faecal culture and gram stain before treatment and weekly during therapy. Further studies will include more detail in this area.

## Conclusion

This hospital-based study found that, of 5 antibiotics compared, doxycycline was the most effective antibiotic in treating chlamydiosis in wild koalas and that chloramphenicol was the next best alternative. Although antibiotic sensitivity testing before treatment is desirable, for *Chlamydia*, cell culture is required. An important additional consideration in antibiotic choice is the gastrointestinal side effects related to the koala’s unique biology, which must be managed during any broad-spectrum antibiotic therapy. The long-term and widespread use of antibiotics in rehabilitated wild animals may lead to antibiotic resistance and may be responsible for changes in observed clinical success of antibiotics over the last three decades where koalas have been treated and released to the wild.

Neither natural infection nor treatment of clinical disease with antibiotics produces any protection from repeat infection. Chlamydiosis remains a major threatening process for koalas, particularly when populations also face ongoing habitat fragmentation and destruction and loss of genetic diversity. Vaccination of young koalas prior to infection may improve the prospects for this species. Only habitat protection, restoration and connection will allow the survival of the existing koala population, and lead to an improvement in genetic diversity and potentially a reduction in disease susceptibility to conserve koalas in the long term.
